# Investigation of Recycled Expanded Polyamide Beads through Artificial Ageing and Mechanical Recycling as a Proof of Concept for Circular Economy

**DOI:** 10.3390/polym16121730

**Published:** 2024-06-18

**Authors:** Sören Handtke, Lena Brömstrup, Jörg Hain, Fabian Fischer, Tim Ossowski, Sven Hartwig, Klaus Dröder

**Affiliations:** 1 Volkswagen AG, Berliner Ring 2, 38440 Wolfsburg, Germany; 2 Institute of Joining and Welding, Technische Universität Braunschweig, Langer Kamp 8, 38106 Braunschweig, Germany; 3 Institute of Machine Tools and Production Technology, Technische Universität Braunschweig, Langer Kamp 19b, 38106 Braunschweig, Germany

**Keywords:** bead foam, expanded polyamide, material properties, recycling, circular economy

## Abstract

Car manufacturers are currently challenged with increasing the sustainability of their products and production to comply with sustainability requirements and legislation. One way to enhance product sustainability is by reducing the carbon footprint of fossil-based plastic parts. Particle foams are a promising solution to achieve the goal of using lightweight parts with minimal material input. Ongoing developments involve the use of expanded particle foam beads made from engineering plastics such as polyamide (EPA). To achieve this, a simulated life cycle was carried out on virgin EPA, including mechanical recycling. The virgin material was processed into specimens using a steam-free method. One series was artificially aged to replicate automotive life cycle stresses, while the other series was not. The mechanical recycling and re-foaming of the minipellets were then carried out, resulting in an EPA particle foam with 100% recycled content. Finally, the thermal and chemical material properties were comparatively analysed. The study shows that the recycled EPA beads underwent polymer degradation during the simulated life cycle, as evidenced by their material properties. For instance, the recycled beads showed a more heterogeneous molecular weight distribution (an increase in PDI from two to three), contained carbonyl groups, and exhibited an increase in the degree of crystallization from approximately 24% to 36%.

## 1. Introduction

Sustainability has become a key focus for society and industry due to the increasing scarcity of resources and the impact of climate change. As a result, the automotive industry is faced with the challenge of transforming its products and production to meet the resulting requirements and regulatory requirements. Furthermore, reducing the CO_2_ footprint caused by fossil-based plastic components is a crucial starting point. In addition to reducing the use of plastic materials, recycling and reusing them also contribute to this goal. For instance, a current proposal by the European Commission suggests that by 2030, newly registered cars should contain 25% recycled plastics, of which a quarter should be post-consumer recyclate (PCR) [1]. Particle foam materials are a promising alternative to non-foam plastics due to their potential to reduce plastic usage. These materials are composed of particle foam beads, which can contain up to 95% enclosed air. Additionally, they are made of thermoplastics, making them easily recyclable.

Particle foam materials are widely used in the automotive sector due to their high lightweight design potential and insulating and energy-absorbing properties. They serve as pedestrian impact protection (bumper cores) and air guide parts in the exterior area, and as toolboxes in the interior area. Additionally, trays made of particle foam beads are used in production and logistics to deliver components to the production line. Expandable polystyrene (EPS) and expanded polypropylene (EPP) are commonly used for this purpose. Foam part densities in automobiles typically range from 15 to 350 g/L, allowing for high lightweight design potential [2,3]. Particle foam parts are primarily characterised by their good compression properties [4].

Particle foam components can make a versatile contribution to future automotive concepts based on alternative propulsion systems. They can contribute to the energy-efficient air conditioning of the passenger compartment and have a positive effect on the thermal budget of the vehicle interior due to the thermal insulation effect of the closed-cell foam structure. This reduces the energy required for air conditioning, leaving more energy available for the propulsion of the vehicle.

On the other hand, as mentioned above, particle foam components are used as safety-relevant crash components. Their generally good compressive strength makes their use in other crash-relevant areas or structures, such as battery protection, conceivable. Here, increased safety requirements in the form of mechanical and thermal properties must be fulfilled. For these reasons, efforts are being made to develop particle foam beads made of technical polymers such as polyamide (PA). These plastics are characterised, among other things, by higher thermal stability than standard plastics, which enables them to be used in thermally stressed components. In the field of particle foam beads, research has been carried out in recent years/decades on particle foam material made from engineering plastics and is commercially available as expanded polyethylene terephthalate (EPET), for example. Other materials, e.g., expanded polyamide (EPA), were patented for the first time by BASF SE in 2011 [5].

The processing of expanded particle foam beads is currently carried out in a well-established process using a steam-based technology. The steam-based process is characterised by a hollow chamber tool design and the use of steam for the temperature control of the mould and for welding the particle foam beads. The steam pressure curve exhibits an exponential behaviour according to the temperature-dependent gas pressure of water so that a steam pressure of approx. 13 bar is required for temperatures of approx. 190 °C. Using water vapour as an energy carrier medium, the temperature required for welding the particle foam beads is transported into the moulded part [6,7,8,9]. Current research is focusing on the steam-reduced or steam-free processing of particle foam beads. In the field of steam-free processing technologies, radiation-based concepts using dielectric heating of plastics by applying electromagnetic waves, such as microwaves or radio frequency, are being investigated [10,11,12,13]. Another possibility is the variothermal mould temperature control, in which a bead bond is generated by thermal conduction. Such a mould technology is also used in this work.

The general recyclability of the most common polymer foams has been investigated in various studies. For example, Shojaei et al. presented several studies that focused on the recycling of polyurethane foam either by mechanical or chemical recycling [14]. However, the recycling of particle foams has been investigated far less frequently. According to Noguchi et al., the recycling of expanded polystyrene (EPS) can be achieved by compaction and subsequent utilisation as a raw material for consumer goods. Alternatively, chemical recycling or thermal utilisation can be employed [15]. An investigation into the closed-loop recycling of EPS using the dissolution technique was carried out by Mumbach et al., with the result that the chemical, thermal, and rheological properties could be retained [16]. With regard to the impact of the ageing stress on the mechanical properties of EPS and EPP, a study by Weingart et al. indicates that compression properties decline following ageing, whereas flexural properties exhibit an increase [17].

The sustainable use of plastic parts is a major issue, particularly in the automotive industry. One approach is to reprocess and recycle polymer waste to achieve a closed-loop material cycle. Using the automotive industry as an example, this involves meeting the above-mentioned potential legislative requirements. The automotive industry faces the challenge of maintaining the material and mechanical properties of recycled plastic components over a long period of time, as the average life expectancy of a car is around 10 to 15 years. This is especially important in the case of post-consumer recyclates, which can be recycled twice or even three times over a period of 30 to 45 years. The effect of ageing on polyamide particle foam and subsequent re-foaming is of great interest and has not yet been investigated. The following section presents the most important effects and results on the material properties of recycled injection moulded polyamide.

## 2. Scientific Background

The current state of research on EPA is limited due to its ongoing industrial development and the lack of commercial applications. Yeh et al. were able to produce a particle foam based on polyamide 6 (PA 6) using a two-stage batch foam process with CO_2_ as the propellant gas, resulting in a double melting peak typical of the process [18]. In a separate study, polyamide 12 (PA 12) was transformed into EPA through an underwater pelletising process that utilised supercritical CO_2_ and ethanol as a blowing agent [19]. The authors were able to demonstrate that the particle foam beads produced using EPA exhibited superior mechanical and thermal properties when compared to those produced using EPP [20]. 

As thermoplastics, polyamides are generally considered to be mechanically recyclable as they can be converted to a molten state that allows them to be re-formed. However, the feasibility of this process and the quality of the recyclate are limited by the degradation that the polymer undergoes during its previous life cycle and mechanical recycling. This degradation can be caused by factors such as (UV) radiation, and thermal and mechanical stresses. Degradation can result in various consequences such as the shortening of polymer chains, cross-linking, or the formation of functional groups [21,22].

As there is limited literature on EPA, there are currently no empirical data available on recycling this material, especially for producing re-expanded beads. However, commercial polyamides developed for processing in the injection moulding process have been extensively investigated in the past regarding their degradation and can serve as a basis here. 

Pliquet et al. investigated the thermal degradation of polyamide 6.6 in a ventilated oven at temperatures up to 200 °C. The thermo-oxidative degradation is based on three chemical mechanisms, resulting in three characteristic peaks at 1756, 1734, and 1712 cm^−1^ in the carbonyl region of an FTIR measurement [23]. Deshoulles et al. (using a PA 6) demonstrated that thermo-oxidative ageing can cause a reduction in the number average ${\bar{M}}_{n}$ and broaden the molecular weight distribution. The presence of water significantly accelerates this process [24]. 

**Figure 1:**
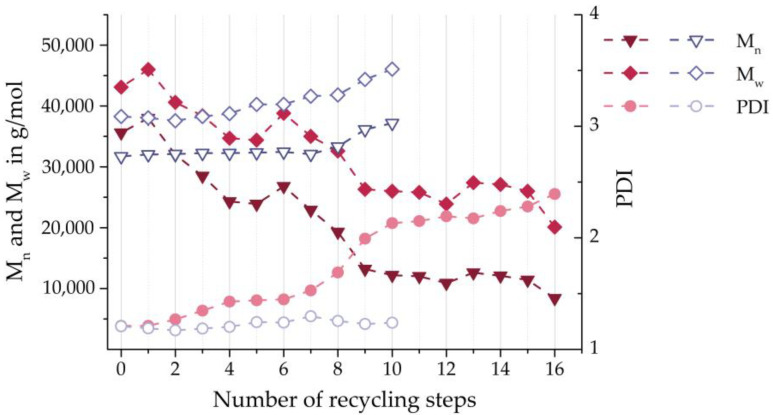
Mean molecular weights and PDI of virgin and recycled PA 6 according to Su et al. [25] (solid symbols) and Lozano-González et al. [26] (hollow symbols).

Su et al. [25] and Lozano-Gonzáles et al. [26] investigated the multiple material recycling of PA 6 using similar injection moulding processes that were repeated up to 16 times. Despite the almost identical procedure, the molecular weight distributions developed in these two studies differed. For instance, Su et al. reported ${\bar{M}}_{n}$ and ${\bar{M}}_{w}$, while the polydispersity index (PDI) increased. In contrast, Lozano-Gonzáles et al. found an increasing trend for the mean molecular weights and an almost stable PDI (see Figure 1). This suggests that chain scission was almost exclusively the degradation mechanism in the former, while the cross-linking mechanism was more likely in the latter [27].

The crystallisation of mechanically recycled polyamides is affected by the “memory effect”. This results in high crystallisation rates at elevated temperatures and is based on hydrogen bonds, which create ordered areas in the molten polymer and act as crystallisation nuclei [25,28]. The melting behaviour of the polyamide recyclate can be influenced by the formation of a bimodal molecular weight distribution, which leads to two melt peaks in DSC thermograms. However, this effect can also be attributed to the formation of γ and α crystallites, which have different melting temperatures [25,29]. In principle, the mechanical recycling of polyamides is possible. However, this process is accompanied by changes in the polymer structure and material properties. The aim of this study is to determine the feasibility of mechanical recycling and the circular use of a polyamide-based particle foam.

## 3. Materials and Methods

**Figure 2:**
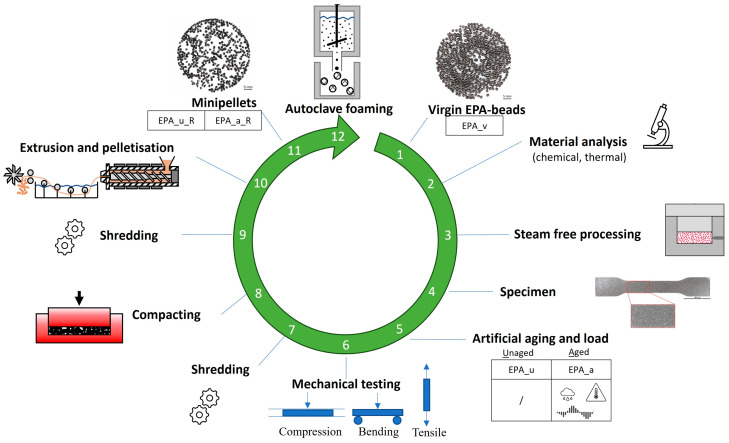
Artificial life cycle with process steps and position of analyses.

As part of this study, polyamide particle foam specimens were subjected to a simulated life cycle with and without artificial ageing; see Figure 2.

Two series were created and exposed to either no ageing or combined ageing, which included changes in ambient temperatures and humidity as well as exposure to vibrations to mimic the influence of climate and a vehicle’s driving profile, respectively. The objective of this study was to analyse the impact of mechanical recycling and re-foaming, as well as internal and external ageing factors, on the material properties of particle foam parts during their life cycle in the automotive sector. The study analysed the material properties of the virgin EPA beads, minipellets after mechanical recycling, and re-foamed EPA beads out of 100% recycled content. The subsequent sections will explain the individual process steps of the artificial life cycle.

### 3.1. Materials

The material used for the investigations in this work was EPA particle foam beads (EPA_v). The beads have a bulk density of around 240 g/cm^3^ and an average diameter of 1.5–2.5 mm.

### 3.2. Steam-Free Processing and Artificial Ageing

**Figure 3:**
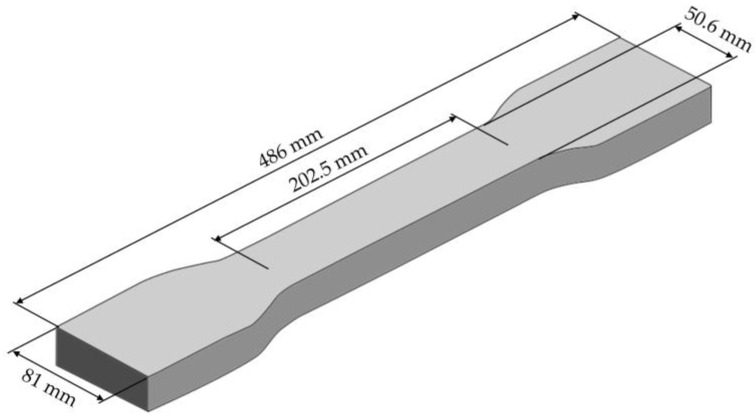
Dimensions of processed EPA specimen.

The specimens were produced from the EPA particle foam beads using a steam-free, variothermal mould foaming process in a temperature range between 189 and 192 °C. The geometry of the specimen is based on the tensile specimen geometry described in ISO 527-2 [30] and is scaled up to enable the production of the specimens from the particle foam beads. The dimensions of the specimens are shown in Figure 3.

The specimens produced were divided into two series in order to be able to differentiate between the influences of ageing effects; see Table 1. The first series was not subjected to any artificial ageing (EPA_u). The second series (EPA_a) underwent a combined ageing process consisting of a preceding climatic cycling in a convection oven and a subsequent high-frequency load, including temperature cycling. The climatic cycling load consisted of a two-part, twelve-hour cycle requiring temperatures of both 80 °C and 40 °C. During the first six hours, the humidity was controlled at 80% relative humidity, while it remained uncontrolled for the second six hours of the cycle. Additionally, a high-frequency load was applied to simulate the effect of vibration. In this test, the specimens were subjected to a test rig for 36 h at frequencies between 5 and 200 Hz.

### 3.3. Mechanical Recycling

In the following step, the specimens were recycled to produce minipellets necessary for the subsequent foaming process. The recycling procedure is based on a real production-related plastics recycling process, therefore, the material was not dried beforehand. The specimens were shredded with a granulator using a 3 mm sieve, compacted, and shredded again under the same conditions. The polymer strands were extruded using a twin-screw extruder at a die temperature of 220 °C. Subsequently, the melt strand, which had a diameter of 3 mm at the nozzle, was drawn through a 3-metre-long water bath for cooling. The pelletiser then drew in the melt strand, thereby reducing it to a diameter of approximately 1 mm before cooling. Finally, the rotating cutter granulated the melt strand into minipellets with a length of about 1 mm. The unaged specimens (EPA_u) and those that have undergone subsequent ageing (EPA_a) were processed separately, resulting in two minipellet batches (PA_u_MP and PA_a_MP), each of which originates 100% from the recycled specimens.

### 3.4. Autoclave Foaming of Recycled Beads

After mechanical recycling, the particle foam beads were produced again from the recycled PA minipellets using an autoclave batch foaming process. The minipellets were fed into a stirring autoclave filled with water and surfactants and saturated with a propellant gas (CO_2_) at 130 °C and 75 bar for 30 min. The expansion of the minipellets into recycled particle foam beads was then initiated through a pressure and temperature drop by opening the valve. The two minipellet batches were foamed separately, resulting in two batches of recycled beads (EPA_u_R = recycled EPA beads of the unaged specimen; EPA_a_R = recycled EPA beads of the aged specimen).

### 3.5. Material Properties

The chemical and thermal properties of the three material stages (virgin bead, minipellet, and recycled bead) were analysed to investigate the effects of ageing, recycling, and foaming. For this purpose, size exclusion chromatography (SEC) and Fourier-transform infrared spectroscopy (FTIR) measurements were conducted, which focus on the chemical structure of the chain molecules, and differential scanning calorimetry (DSC) and thermomechanical analysis (TMA) measurements, which analyse the thermal properties. To ensure statistical accuracy, each analysis method was performed three times per series. The virgin beads and minipellets were not dried, while the recycled beads were dried for 24 h at 80 °C after foaming to remove the water absorbed during the autoclave process.

The SEC measurements were conducted to determine the molecular weight distribution, as well as the number average (${\bar{M}}_{n}$) and weight average (${\bar{M}}_{w}$). The polydispersity index (PDI) was calculated using Equation (1). A PG-IR PolymerChar htgpc from PSS Polymer Standards Service GmbH (Mainz, Germany) with two separation columns was used, with 1,1,1,-3,3,3-hexafluoro-2-propanol (HFIP) as the solvent at a flow rate of 1 mL/min and a temperature of 30 °C.

(1) $$PDI=\frac{M_{w}}{M_{n}}$$

Equation (1): The calculation of polydispersity (PDI)

To determine whether oxidative degradation processes have occurred, infrared (IR) spectra can be used to identify the presence of functional groups such as carbonyl groups in a sample. In this study, the measurements were conducted using attenuated total reflectance (ATR)-FTIR from Bruker (Billerica, MA, USA) in the infrared spectrum between 4000 and 600 cm^−1^. The resolution was 4 cm^−1^ with 32 scans.

The melting and crystallisation behaviour of the samples was investigated by the DSC measurements using a NETZSCH DSC 204 F1 Phoenix (Selb, Germany). The temperature range of −20–220 °C was used to record the first and second heating curves and the intermediate cooling curve. The heating/cooling rate was 10 K/min and nitrogen was used as the purge gas. The degree of crystallinity $X_{c}$ was determined according to Equation (2). ${∆\;H}_{m}$ is the measured melting enthalpy of the polymer used and ${∆H}_{100\%}$ = 190 J/g is the melting enthalpy of 100% crystalline polyamide.

(2) $$X_{c}=\frac{{∆\;H}_{m}}{∆\;H_{100\%}}*100\%$$

Equation (2): The calculation of degree of crystallinity $X_{c}$

Single-bead TMA measurements were conducted to analyse the thermal stability of the virgin and recycled particle foam beads. For this purpose, a TMA 402 F1 Hyperion from NETZSCH was used. A single bead was placed under the glass plunger and loaded with a measuring force of 0.01 N. The temperature was held isothermally at 10 °C for 15 min to obtain a stable initial length. Subsequently, the temperature was increased to 205 °C at a heating rate of 5 K/min.

## 4. Results and Discussion

**Figure 4:**
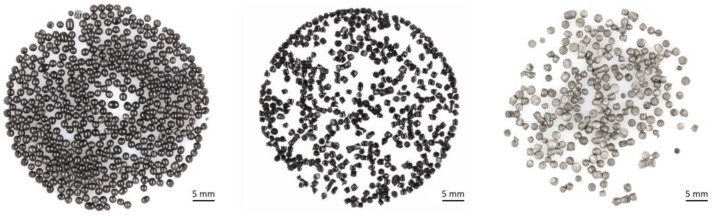
Photographs of three stages of polyamide material that were analysed: virgin expanded polyamide (EPA) particle foam beads (**left**), minipellets of PA after recycling (**middle**), and recycled EPA particle foam beads (**right**).

Figure 4 shows the three stages of the polyamide material on which the material properties were analysed. The recycled EPA beads are significantly greyer than the virgin material and the minipellets. This difference in colour may be attributed to the stretching of the polymer chains during the expansion process or to the degradation of the coloured particles during ageing and recycling.

### 4.1. Change in Chemical Properties

The chemical properties of the three material grades (virgin beads, minipellets beads, and recycled beads) were characterised using the SEC and FTIR analysis, and thermal properties were determined using the DSC and TMA analysis.

**Figure 5:**
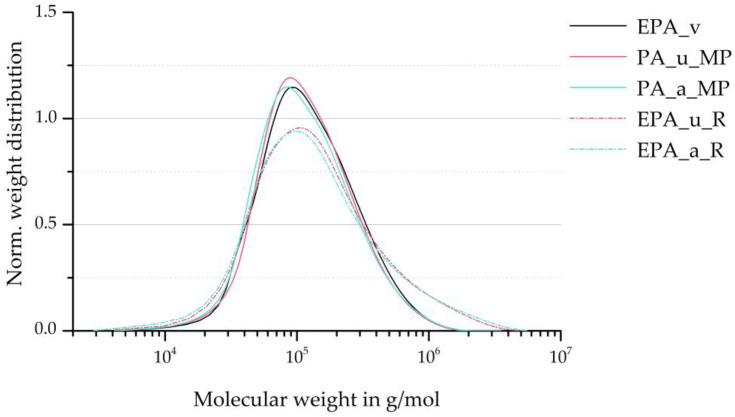
SEC curves of virgin EPA beads, minipellets, and recycled EPA beads of differentially aged specimens.

**Figure 6:**
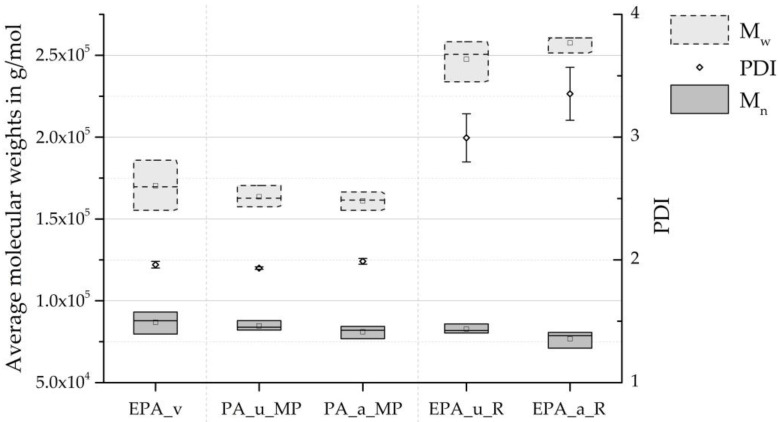
Average molecular weights and PDI of virgin material (_v), minipellets (_MP), and recyclate beads (_R) by SEC.

The SEC curves are presented in Figure 5. Depicted are the representative curves of the three material grades, divided according to ageing. Figure 6 compares the number average ${\bar{M}}_{n}$, weight average ${\bar{M}}_{w}$, and polydispersity indices. It can be observed that the number average ${\bar{M}}_{n}$ decreases only slightly from the virgin material to the minipellets to the recycled beads. Similarly, the weight average ${\bar{M}}_{w}$ changes only slightly towards lower molecular weights as a result of material recycling. However, the EPA_u_R and EPA_a_R series demonstrate that the autoclave foaming process, which occurs between the minipellets and the recycled beads stages, results in a substantial increase in ${\bar{M}}_{w}$.

The weight average is commonly used to indicate the rheological properties of a polymeric material. The observed increase in ${\bar{M}}_{w}$ may suggest an increase in melt viscosity. Additionally, the PDI increases from about two for the virgin EPA and the minipellets to about three for the recycled beads, indicating a broader molecular weight distribution and more non-uniform chain molecules. In summary, the autoclave foaming process significantly affected the molecular weight distribution, while material recycling had only a minor influence in comparison.

The change in molecular weight distribution after the autoclave process may be due to the high mechanical stress on individual polymer chain segments during expansion. Before opening the autoclave and allowing the minipellets to expand into the particle foam beads, the temperature was maintained just below the melting point of the PA. By taking up CO_2_ molecules, the melting point temperature was lowered. This achieves a high degree of crystallisation without plasticising the polymer, as the polymer chains retain sufficient freedom of movement. The remaining long-chain molecules exist in amorphous segments, where multiple chains may be entangled. Furthermore, hydrogen bonds are formed between many of the amide groups in polyamides [31].

Table 2 presents the average molecular weights and PDIs of the virgin EPA beads and the recycled beads and the values of the recycled material in the references presented in Section 2. This shows that the decrease in $M_{n}$ observed in this work after the first recycling cycle differs from the development of $M_{n}$ in the literature. Furthermore, the increase in $M_{w}$ by 45.36% and 51.23% for EPA_u_R and EPA_a_R, respectively, also deviates significantly from the trend of the studies cited. The significant decrease in Mn and increase and Mw ultimately caused a strong change in the PDI in this work, which was not found by Su and Lozano, as the change in $M_{n}$ and $M_{w}$ is correspondingly lower. As described above, the autoclave process contributed significantly to the change in molecular weight distribution in this study. The change in the listed characteristic values is correspondingly lower in the comparative literature cited, as only an injection moulding process and no foaming was carried out.

**Figure 7:**
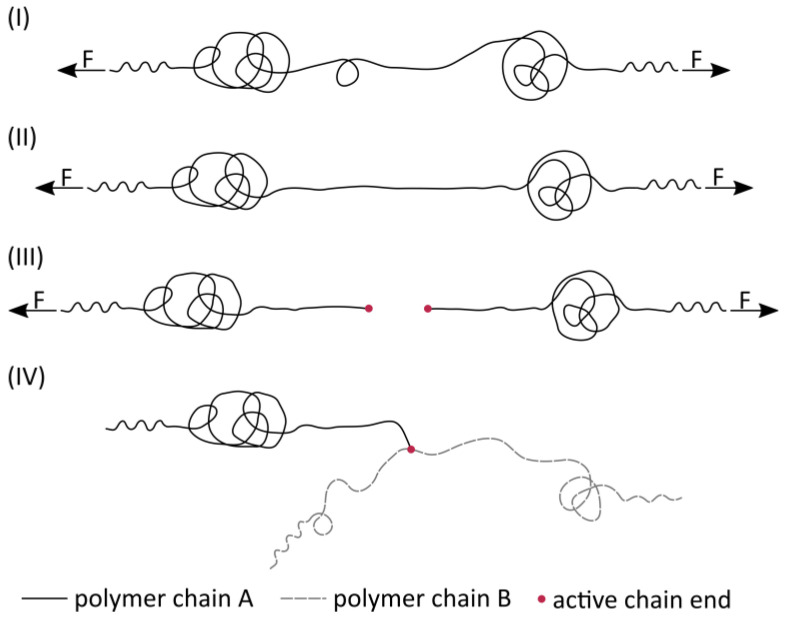
A possible mechanism for chain extension during autoclave foaming is depicted in schematic form. (**I**: Streched polymer chain, **II**: Middle segment is fully stretched, **III**: formation of two active chain ends, **IV**: forming of branched polymer chain).

The listed mechanisms counteract the expansion caused by the phase separation between the polymer and CO_2_ by restricting chain mobility. Figure 7 schematically shows that this could initially lead to the stretching of individual chain segments (II) and subsequently to the overstressing of molecular bonds (III), which then split and create two active chain ends. The continued expansion could cause the chain ends to move away from each other and form branches on other chain molecules (IV). Chain branching has a significant impact on the rheological properties of polymers. It restricts the movement of chains against each other, resulting in altered properties.

The molecular bonds experience greater mechanical stress as chain length increases and distance from the chain centre decreases. Therefore, chain scissions are more likely to occur in long polymer chains near their chain centre [19]. If the active chain end reacts with a random chain link of another nearby polymer chain, this second chain’s (chain B in Figure 7) molecular weight is on average ${\bar{M}}_{w}$. This process can be understood, for example, in the study by Liu et al. [32]. Since chain scissions primarily occur within longer polymer chains and the reaction of an active chain end with an already long polymer chain is more probable, an average lengthening of the already long polymer chains is to be expected. This is consistent with the relationship observed between the minipellets and the recycled beads in Figure 5 and Figure 6.

**Figure 8:**
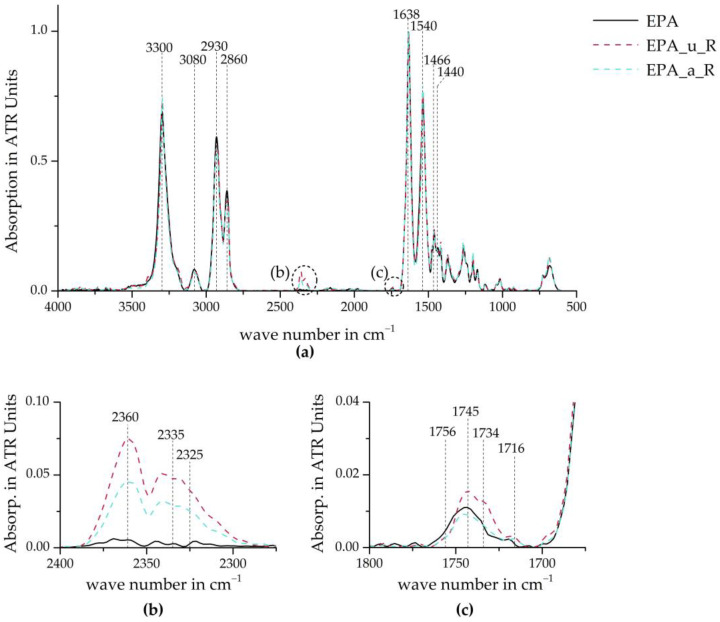
ATR-FTIR absorption of virgin EPA and recyclate beads. (**a**) Whole spectrum; (**b**) CO_2_ absorption bands; (**c**) carbonyl region.

Figure 8 compares the ATR-FTIR curves of the three material stages. The entire spectra (see Figure 8a) show no significant change in peaks resulting from the recycling or re-foaming of the minipellets into the recycled beads, which would indicate a large-scale change in molecular structure. Figure 8b shows absorption peaks indicating CO_2_ in all the samples except the EPA virgin material [33]. This could be due to CO_2_ from the ambient air, as explained above. However, it is possible that small amounts of CO_2_ exist between the polymer chains, as CO_2_ can be a degradation product of PA 6.6. Additionally, CO_2_ is used to foam the minipellets into the recycled beads.

In the carbonyl region of the IR spectrum (Figure 8c), clear peaks are visible in both the virgin material and the recycled beads. However, only noise is recognisable in the minipellets, and it cannot be clearly assigned to any of the peaks. Table 3 summarises the band assignment of the highlighted wavenumbers. The C=O elongation at 1745 cm^−1^ is most likely due to the bond already present in PA 6.6. However, the presence of carboxylic acids and imides indicates the thermo-oxidative degradation of the material. These peaks were also cited in studies by Deshoulles et al. [24] and Pliquet et al. [23] as the evidence of polyamide degradation. It cannot be assumed that the recognisable difference between EPA_u_R and EPA_a_R indicates a lower degradation of the latter series. It can be stated that both EPA_u_R and EPA_a_R exhibit more pronounced peaks, indicating material degradation throughout the entire recycling process, from bead to bead. It is unclear whether this degradation occurred during the simulated life cycle, the mechanical recycling cycle, or the re-foaming process due to the insufficient measurement resolution of the EPA_u_MP and EPA_a_MP series.

### 4.2. Change in Thermal Properties

**Figure 9:**
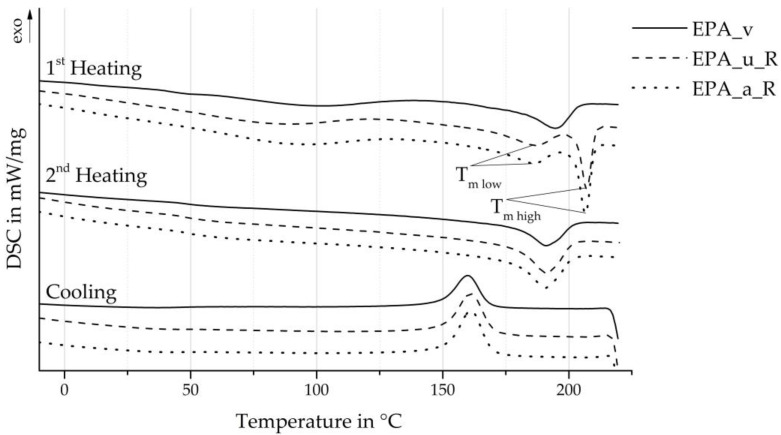
DSC curves of virgin material and recyclate beads.

Figure 9 shows the melting and crystallisation behaviour of the virgin and recycled particle foam beads, as determined by the DSC curves. The particle foam beads made from recycled material exhibit a double melting peak with the melting temperatures $T_{m\;low}$ and $T_{m\;high}$, which is typical for beads produced by autoclave foaming. This bimodal melting behaviour results from two mechanisms [34]. On one hand, the existing crystallites are perfected at the temperature in the autoclave close to the melting point. This results in the formation of high-melting crystal structures corresponding to $T_{m\;high}$. On the other hand, the less perfect crystallites melt during the saturation time, which recrystallises during the expansion and forms crystallite fractions corresponding to $T_{m\;low}$. The melting and crystallisation temperatures determined from the DSC measurements are summarised in Table 4. There is a slight difference between $T_{m\;low}$ and $T_{m\;high}$ in the EPA_u_R and EPA_a_R series. This difference is most likely due to deviations in the process control during the autoclave process.

In contrast to the differences observed between the virgin material and recycled beads during the first heating process, the second DSC heating curves and the cooling process curves exhibit remarkable similarities. This observation is further supported by the average melting and crystallisation temperatures, which were determined from the DSC measurements and are summarised in Table 4. The second heating process provides information on the basic material behaviour since the thermal history of a plastic is reset during the first DSC heating process. This material behaviour was not significantly influenced by the material recycling and foaming into the particle foam beads within the scope of these investigations.

The DSC cooling process shows a crystallisation peak temperature approximately 1 °C higher for the EPA_u_R and EPA_a_R samples compared to the virgin EPA or the minipellets. This suggests that stable crystal structures can be produced at slightly higher temperatures through crystallisation. The presence of impurities in the polymer, introduced during the recycling or foaming process, may be a contributing factor. Additionally, polyamides can exhibit a phenomenon known as “memory effect” (see Section 2), whereby the crystalline structure melts during a heating process but some hydrogen bonds persist. These hydrogen bonds form ordered regions within the melt which act as crystallization nuclei, resulting in higher crystallisation temperatures and rates [32].

A further distinction between the virgin and recycled beads can be observed with regard to the crystallisation enthalpy $H_{c}$. The unaged recycled beads (EPA_u_R) exhibit an increase in the crystallisation enthalpy of approximately 28%, while the aged recycled beads (EPA_a_R) demonstrate an increase of approximately 32.5%. One potential explanation for this observed increase is the presence of impurities derived from the recycling process. These impurities may have served as nucleation sites for the observed crystallization.

**Figure 10:**
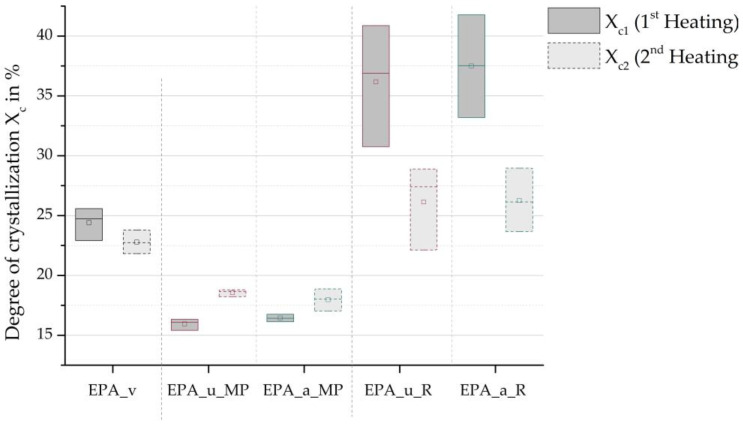
Degree of crystallisation of virgin material, minipellets, and recyclate beads.

Figure 10 shows a box diagram of the degrees of crystallisation obtained from the DSC heating curves. The degree of crystallisation obtained from the initial heating process is primarily dependent on the polymer’s thermal history. The degree of crystallisation in the EPA virgin material is lower due to the foaming process used by the manufacturer compared to the autoclave foaming process used in these investigations. However, both foaming processes result in a higher degree of crystallisation than the material would achieve under controlled DSC conditions. This is evident from the fact that $X_{c1}$ is greater than $X_{c2}$ in the EPA_v, EPA_u_R and EPA_a_R series. Additionally, it can be concluded that the foaming process used by the virgin material manufacturer leads to a more uniform degree of crystallisation than the autoclave foaming process, as indicated by the size of the boxes. The level of crystallisation that takes place during extrusion in the process of material recycling is considerably lower than the values found for the particle foam beads. This is because the melt strand was pulled through a water bath after extrusion, causing it to cool rapidly.

The possibility that the “memory effect” influenced the crystallisation behaviour during the DSC measurement is supported by the mean degrees of crystallisation shown in Figure 10. The mean degree of the crystallisation of the recycled beads was higher than that of the virgin material during the second heating process, despite the first heating process resetting the thermal history. It is evident that the high standard deviation in the particle foam beads made from recycled material was maintained during the second heating process. This leads to the conclusion that some information regarding the previous crystallite structure was preserved.

**Figure 11:**
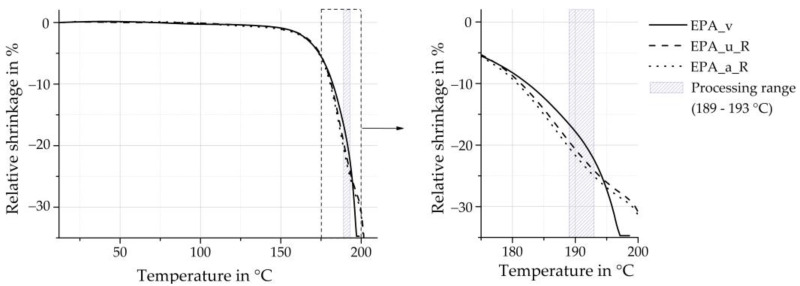
Relative shrinkage of single virgin and recyclate beads.

Thermal stability in the processing area was determined using the TMA measurements. Figure 11 (left) shows the curves of the virgin EPA material compared to the recycled particle foam beads against temperature. It can be seen that the curves are the same up to about 175 °C. Above this temperature, the negative change in the length of the virgin material increases exponentially. The length change curve for the recycled beads is also almost exponential, but shows a step at 195 °C caused by the double melting peak. Due to this difference between the TMA curves of the virgin material and the recycled beads, the negative change in the length of the recycled beads is higher up to 195 °C (see Figure 11 (right)). Above this temperature, the negative change in the length of the virgin material is higher. The recycled beads, therefore, remain thermally stable to a higher temperature than the virgin EPA material analysed due to the autoclave process. A comparison with minipellets is not made at this point as the thermal stability of minipellets in the TMA test does not allow conclusions to be drawn about changes in properties due to material recycling or ageing.

The processing temperature range shrinkage is especially significant for the moulding process. For the lower processing temperature of 189 °C, the average shrinkage of the particle foam beads made from recyclate is 19% greater for EPA_u_R and 23% greater for EPA_a_R compared to the shrinkage of the virgin EPA material. At the upper end of the processing range (193 °C), the shrinkage of the recycled beads is still higher than that of the analysed virgin material, but the difference has decreased to approximately 10%.

There may be two reasons for this behaviour. On the one hand, the degradation of the material described through the carboxylic acids and imids, described by the FTIR measurements above. Or on the other hand, by the inner cell structure. The inner cell structure can play an important role with regard to the mechanical stability of the material under thermal load. The formation of the inner cell structure, including the homogeneity of the size of the inner cells, the ratio of open vs. closed cell content, the inner and outer wall thickness, and other factors, can be decisive in this regard. This assumption can be examined by using scanning electron microscopy (SEC).

## 5. Conclusions

The investigations in this study have revealed that the simulated life cycle, as well as the subsequent mechanical recycling and re-foaming of the minipellets into particle foam beads, resulted in polymer degradation. This is evidenced by the formation of carbonyl groups, which are indicative of thermal-oxidative ageing and were detected in the recycled beads using the ATR-FTIR measurements.

The analysis of the molecular weight distribution and average molecular weights indicates that simulated ageing and mechanical recycling have only a minor impact on them. In contrast, the re-foaming process using the autoclave method has a significantly greater effect on the polymer chains. The expansion of the minipellets into the particle foam beads causes a shift in the molecular weight distribution towards higher chain lengths. As a result, the PDI increases from around two (for virgin material and minipellets) to three (for recycled beads). This could be attributed to chain scissions occurring near the centre of the chain due to sudden mechanical stress on the polymer chains. The active chain ends resulting from this process can form branches on neighbouring polymer chains, increasing the weight average ${\bar{M}}_{w}$ of the plastic. This affects the rheological properties of the PA, as the branching prevents the chains from sliding against each other and can also hinder crystallisation. To partially counteract the trend of increasing molecular weights in subsequent recycling cycles, one possible approach would be to add low molecular weight PA, for example, from a previous recycling cycle. However, this approach does not fully counteract the mechanism of chain elongation induced by chain scission in the autoclave process.

One alternative method would be to decrease the mechanical stress on the polymer chains while expanding. Polymer materials exhibit time-dependent mechanical behaviour due to the sliding movement of chain molecules against each other. Therefore, a slower expansion process, such as through multiple pressure stages, may be beneficial. The thermal analyses indicate that mechanical recycling and foaming through an autoclave process have impacted the material properties relevant to processing. These effects can be observed in the first DSC heating process. The investigations demonstrate that the process control during mechanical recycling resulted in minipellets with relatively uniform thermal properties and a low degree of crystallisation. This outcome is advantageous for the subsequent autoclave process. In this work, the process produced particle foam beads with a characteristic double melting peak and higher crystallinity compared to the virgin material.

In a second recycling cycle of the recycled beads, it is important to investigate the relevance of degradation at the molecular level. The altered molecular weight distribution may require different process parameters during extrusion in the mechanical recycling process, due to the increased viscosity, in order to enable the renewed production of minipellets. Adjustments to the process may be required during the re-foaming step in the autoclave process due to changes in average molecular weights, molecular structure, and associated chain convolutions. If this trend persists in subsequent recycling cycles, it could pose challenges for process control in the production of recycled beads and negatively impact the quality of particle foam beads made from recycled material.

## Figures and Tables

**Table 1:** Overview of processed series and different ageing.

Load/Aging Type	Series 1	Series 2
Climate changing	/	✔
High frequency	/	✔
Abbreviation	EPA_u	EPA_a

**Table 2:** Average GPC data from this work and the selected literature. The values for the source Su et al. are estimated.

	This Work			Su et al. [25]		Lozano-Gonzalez et al. [26]	
	EPA_v	EPA_u_R	EPA_a_R	Virgin PA	PA Recycled Once	Virgin PA	PA Recycled Once
$M_{n}$ [g/mol]	86,870	82,675 (−4.83%)	76,812 (−11.58%)	35,600	38,000 (+6.74%)	31,750	32,000 (+0.79%)
$M_{w}$ [g/mol]	170,300	247,540 (+45.36%)	257,550 (+51.23%)	43,100	46,000 (+6.73%)	38,250	38,000 (−0.65%)
PDI	1.96	3.00 (+52.73%)	3.36 (+71.04%)	1.21	1.21 (±0%)	1.20	1.18 (−1.43%)

**Table 3:** Band assignments and corresponding wavenumbers of identified peak in carbonyl region.

Species	Wavenumber in cm^−1^	Source
“Free” carboxylic acids	1756	[10,11]
C=O stretching	1745	[12,13]
Imides	1734	[10,11]
“Bonded” carboxylic acids	1716	[11]

**Table 4:** DSC melting and crystallisation temperatures and enthalpies of virgin material, minipellets, and recyclate beads.

	1st Heating			2nd Heating		1st Cooling	
	T_m low_ (°C)	T_m high_ (°C)	Δ H_m_ (J/g)	T_m_ (°C)	Δ H_m_ (J/g)	T_c_ (°C)	Δ H_c_ (J/g)
EPA_v	194.6 ± 0.2		46.39 ± 2.11	191.2 ± 0.5	43.29 ± 1.53	160.0 ± 0.3	36.0 ± 1.51
PA_u_MP	196.0 ± 0.3		30.29 ± 0.74	191.8 ± 0.1	35.27 ± 0.46	160.0 ± 0.1	29.4 ± 0.42
PA_a_MP	196.1 ± 0.2		31.23 ± 0.49	191.8 ± 0.1	34.14 ± 1.44	159.3 ± 0.1	30.1 ± 0.17
EPA_u_R	187.4 ± 0.1	207.0 ± 0.2	68.74 ± 7.90	191.1 ± 0.2	49.66 ± 5.52	160.9 ± 0.1	46.0 ± 5.48
EPA_a_R	186.2 ± 0.2	206.5 ± 0.1	71.24 ± 6.66	190.5 ± 0.2	49.89 ± 4.11	161.1 ± 0.1	47.7 ± 4.24

## Data Availability

The raw/processed data required to reproduce these findings cannot be shared at this time as the data also form part of an ongoing study.
